# Alive and kicking: suicide rates and major soccer events in Austria, Germany and Switzerland

**DOI:** 10.1093/eurpub/ckad042

**Published:** 2023-03-20

**Authors:** Eva-Maria Pichler, Martin Ploederl, Lucas Rainer, Thomas S Gilhofer, Jonathan Michel, Jan Gerrit van der Stouwe, Thomas F Luescher, Christian M Schmied, Wolfram Kawohl, Jens Kronschnabel, David Niederseer

**Affiliations:** Department of Psychiatry and Psychotherapy, Psychiatric Services Aargau AG, Brugg-Windisch, Switzerland; Department of Psychiatry, Psychotherapy and Psychosomatics, University of Zurich, Zurich Switzerland; Department of Inpatient Psychotherapy and Crisis Intervention, University Clinic for Psychiatry, Psychotherapy, and Psychosomatics, Christian Doppler Clinic, Paracelsus Medical University, Salzburg, Austria; Department of Clinical Psychology, Christian Doppler Clinic, University Clinic for Psychiatry, Psychotherapy, and Psychosomatics, Paracelsus Medical University, Salzburg, Austria; Department of Inpatient Psychotherapy and Crisis Intervention, University Clinic for Psychiatry, Psychotherapy, and Psychosomatics, Christian Doppler Clinic, Paracelsus Medical University, Salzburg, Austria; Department of Clinical Psychology, Christian Doppler Clinic, University Clinic for Psychiatry, Psychotherapy, and Psychosomatics, Paracelsus Medical University, Salzburg, Austria; Department of Neurology, Christian-Doppler Medical Centre, Paracelsus Medical University, Centre for Cognitive Neuroscience Salzburg, EpiCARE, Salzburg, Austria; Department of Cardiology, University Heart Center, University Hospital Zurich, University of Zurich, Zurich, Switzerland; Department of Cardiology, University Heart Center, University Hospital Zurich, University of Zurich, Zurich, Switzerland; Department of Cardiology, University Heart Center, University Hospital Zurich, University of Zurich, Zurich, Switzerland; Center for Molecular Cardiology Schlieren Campus, University of Zurich, Schlieren, Switzerland; Royal Brompton & Harefield Hospitals, Imperial College and Kings College, London, UK; Department of Cardiology, University Heart Center, University Hospital Zurich, University of Zurich, Zurich, Switzerland; Department of Psychiatry, Psychotherapy and Psychosomatics, University of Zurich, Zurich Switzerland; Clienia Schlössli AG, Oetwil am See, Switzerland; Department of Psychiatry and Psychotherapy, Psychiatric Services Aargau AG, Brugg-Windisch, Switzerland; Department of Cardiology, University Heart Center, University Hospital Zurich, University of Zurich, Zurich, Switzerland

## Abstract

**Background:**

Major sporting events are postulated to reduce suicide rates by increased social connectedness, by identifying with winning teams, or, conversely, to increase suicide rates by the ‘broken promise effect’.

**Methods:**

In our observational epidemiological study, we investigated changes in suicide rates between 1970 and 2017 in Austria, Germany and Switzerland during the European and World Soccer Championships in general, and on days that the home team played, won or lost.

**Results:**

Combining all three studied nations no statistically significant change in the incidence of daily suicides during soccer championships compared to a control period was noted (38.29 ± 9.02 vs. 37.33 ± 10.58; incidence risk ratio = 1.03; 95% confidence interval: 1.01–1.05, *P *=* *0.05). Essentially, no differences in the expected directions were found, and none remained statistically significant after correcting for multiple comparisons in subgroups for country, age and gender in all three studied countries. Compared to a control period, neither a significant difference in the respective national suicide rate was found after Germany’s four championship victories nor after Austria’s emotional only win over Germany.

**Conclusion:**

Our results do not support the assumption of increased social connectedness and, thus, lowered suicide risk during major sporting events or changes in suicide risk depending on the outcome of important games as predicted by the broken promise effect or changes in self-efficacy by identification with winning teams.

## Introduction

Major sporting events have an enormous social, cultural, economic and political impacts worldwide. For example, up to 48% of the German population viewed the 2014 *Fédération Internationale de Football Association* (FIFA) World Cup final, when Germany defeated Argentina 1–0.^1^ Given the huge emotional impact major sporting events have drawn the focus of suicide research. Several mechanisms have been postulated to explain why major sporting events may impact suicide rates. For example, such events may increase social connectedness and thus reduce suicide risk.^2^^,^^3^ Identification with a sports team can increase perceived self-efficacy; thus, participating in major sports events when one’s own team is winning may reduce suicide rates (or vice versa).^4^^,^^5^ Similarly, the positive expectations associated with major sporting events may reduce suicide rates, but the ‘broken promise’ effect, meaning that a person suffering suicidal ideation will wait for a significant improvement of their circumstances based on a certain event,^6^ may increase suicide risk.^7^

Studies investigating the relationship between suicide rates and major sporting events in hockey, American Football and soccer have yielded inconsistent results. An early study on hockey games in Canada reported no association between the outcome in finals and the national suicide rate.^8^ However, in that study, when the home team lost, there was an increase in suicides among young and unmarried men but not among married men. During the Super Bowl, the championship (CS) game of the National Football League, two studies did not find changes in suicide incidences,^9^^,^^10^ whereas a more recent study by Joiner et al. reported lower suicide rates. Successful regional sports team performance was associated with lower suicide rates in two other studies.^5^^,^^7^ In France during the 1998 FIFA soccer World Cup a reduction in suicide rates was reported both during the entire CS period (−10%), especially following games involving the French team (−20%), among French men (−23%), and 30- to 44-year-old individuals (−33%).^11^ In contrast, in Hungary suicide rates, did not change during the *Union des Associations Européennes de Football* (UEFA)/FIFA soccer championships.^12^ However, this study investigated the CS period in general, and did not focus on Hungarian games.

Overall, therefore, empirical studies on the association between suicide and major sporting events are inconclusive, although some significant associations have been reported for subgroup populations or depending on the outcome of certain sports events. However, most studies have focused on single sporting events or were limited by small sample size. Thus, further research is needed to clarify the nature and robustness of the effect of major sporting events on suicide rates. The present study investigated changes in suicide rates during major soccer championships (FIFA World Cup and UEFA European Championship) in the three German-speaking European countries; Austria, Germany and Switzerland. We expected that suicide rates would decrease during championships in general, and specifically on days that the home team won. In contrast, we expected an increase of suicides on days that the home team lost.

## Methods

In this observational epidemiological study, we investigated changes in suicide rates between 1970 and 2017 in Austria, Germany and Switzerland during the European and World Soccer Championships compared to a reference period.

### Data and variables

#### Suicides

The number of daily suicides from Austria (1 January 1970–31 December 2017), Germany (01 January 1992–31 December 2017) and Switzerland (01 January 1970–31 December 2017) was requested from the corresponding national statistics organizations of all three countries. Statistics Austria (http://www.statistik.at/) and the Swiss Federal Statistical Office (https://www.bfs.admin.ch/bfs/de/home.html) served as data acquisition sources for Austria and Switzerland, respectively. Data from Germany were limited to a shorter timeframe (01-01–2017) for budgetary reasons. Furthermore, the German data were not directly available for analysis. Instead, we had to provide the R-code to the German Federal Statistical Office (Destatis) who then generated the results. A time series of daily suicides was created for each country (Austria, Germany and Switzerland). Furthermore, we created a time series for the subpopulations of men, women, the younger population overall aged 30–44 and young males aged 15–34, based on differential findings reported in the existing literature mentioned above.

#### Championships

Information on soccer championships was retrieved from a publicly available dataset (https://www.kaggle.com/martj42/international-football-results-from-1872-to-2017). The analysis was restricted to European and World championships. Information about games in the later phase of competitions (the so-called ‘knock-out’ games) was manually extracted, based on data retrieved from the official websites of the tournaments at fifa.com and uefa.com (see Supplementary material for details). For better understanding and readability, we chose ‘home team’ in the text as a synonym for the respective country’s national team, independent of the venue.

**Figure 1:**
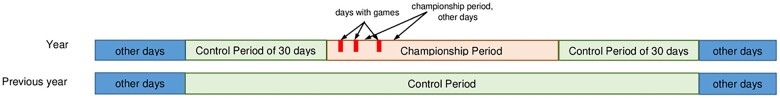
Approach Two—championship phase vs. control period (±30 days in same year and related period in previous year) vs. other days; or days with games vs. control period vs. other days in championship phase vs. other days

A time series with information about the championships was created for each of the three countries. This included information about the time period of a CS, days when the home team was playing, days when the home team won, days when the home team lost and days when the home team took part in a later phase of the CS (i.e. ‘knock-out’ phase). Furthermore, since Germany won the UEFA/FIFA championships seven times, these seven finals were used for a separate analysis (including the next day in a sensitivity analysis). We also analyzed ‘the wonder of Cordoba’, the only time that Austria has defeated Germany, on 21 June 1978. In the time series, as a reference period, we used 30 days before and after the CS period. Additionally, to increase the statistical power, the corresponding periods in the year prior to the CS were also used, similar to related publications.^13^^,^^14^ For example, if the CS took place in June 2000, then the control period encompassed May and July of 2000, as well as May, June and July 1999 (see figure 1).

We consulted the *ethics committee of north-western and central Switzerland (Ethikkommission Nordwest- und Zentralschweiz,* Project-ID Req-2022-00752). Our study was not in the scope of their responsibility because our project is not a study according to the human research law Art. 2, and ethical approval was waived.

#### Statistical analysis

Data were analyzed using Poisson regression models, with the daily number of suicides as dependent variables and with CS periods (or days with games) as independent variables. To account for the seasonal and weekly variation of suicide risk and for long-term trends in suicide,^14^ the regression model included day of the week, month and year. Since annual suicide rates have a non-linear change between 1970 and 2017 (data not shown), effects of the year were modelled using a spline with three knots.

#### Sensitivity analysis

To analyze the days when the national team played, we also included the next day in the sensitivity analyses, as the efficacy effect as well as the broken promise effect may carry over to the next day (as known from research about holidays).^15^^,^^16^ We also ran analyses with unrestricted control periods, that is, using the information from the whole year (and without the previous year as control). As the results of these sensitivity analyses are largely comparable, we provide them as Supplementary material on the Open Science Framework.^17^ We used R 4.1.2. for all statistical analyses.^18^ To reduce the effect of random significant results in a case series with limited events, we applied the Bonferroni–Holm correction method. A *P*-value of less than 0.05 was considered statistically significant, while less than 0.10 was viewed as a trend.

The R-Code will be made public via the OSF (https://osf.io/4b6t5/). The study protocol was not registered. However, for the analysis of the German data, we had to provide the R-Code to the German Federal Statistics Department, who returned the results. This procedure was performed only once. Thus, whereas the statistical analysis for Switzerland and Austria can be considered partly exploratory, the results from Germany can be considered a pre-planned replication.

## Results

### Suicides during the entire CS period

For Germany, Austria and Switzerland combined, the mean (±SD) incidence of daily suicides during championships was 38.29 (±9.02) compared to 37.33 (±10.58) during the control period. Adjusting for day of the week, month and year, this resulted in a non-significant statistical trend towards increased suicide rates during championships [incidence risk ratio (IRR) of 1.03, (95% confidence interval (CI): 1.01–1.05, *P* < 0.1)]. No statistically significant result was revealed in the analysis of each country’s entire study population including in the various subgroup analyses (table 1).

**Table 1:** Incidences of daily suicides during championships vs. control period—all three nations combined

	Austria, Germany and Switzerland combined	
	*M* (SD)	IRR (95% CI)
Total sample		
Control period	37.33 (10.58)	ref
Championship	38.29 (9.02)	1.03 (1.01–1.05)*
Men		
Control period	27.41 (8.09)	ref
Championship	28.03 (6.98)	1.03 (1.00–1.05)
Women		
Control period	10.25 (3.73)	ref
Championship	10.26 (3.86)	1.01 (0.97–1.05)
Aged 30–44		
Control period	8.28 (3.66)	ref
Championship	8.45 (4.18)	1.05 (1.01–1.10)
Men aged 15–34		
Control period	5.23 (2.86)	ref
Championship	5.26 (2.89)	1.02 (0.96–1.07)

*Note:* IRRs are adjusted for day of week, month and year. Analysis was based on data from 1992 to 2017, since data from Germany were only available during this time period.

CS, championship; IRR, incidence risk ratios; ref, reference category.

* *P* < 0.10.

### Days during CS period when the home team played a game

Table 2 describes the incidences of daily suicides on the days that the home team participated in a game during the CS vs. control period vs. other CS days when the respective home team did not play. No statistically significant associations were identified in any country and in any of the subgroups. In Germany, during CS days when the home team did not play a game, there was a non-significant statistical trend toward increased suicide rates (IRR = 1.03; 95% CI: 1.01–1.06, *P *=* *0.076) in the total sample.

**Table 2:** Incidences of daily suicides during days when the home team took part in games vs. control period vs. other championship days

	Austria		Germany		Switzerland	
	*M* (SD)	IRR (95% CI)	*M* (SD)	IRR (95% CI)	*M* (SD)	IRR (95% CI)
Total sample						
Control period	4.76 (2.44)	ref	29.71 (9.79)	ref	3.25 (1.85)	ref
Game with home nation	4.65 (2.77)	0.94 (0.77–1.14)	29.03 (7.57)	1.00 (0.95–1.04)	3.36 (1.99)	1.04 (0.85–1.28)
Other CS days	4.56 (2.26)	0.97 (0.89–1.05)	30.98 (7.57)	1.03 (1.01–1.06)*	3.16 (1.91)	0.99 (0.91–1.08)
Men						
Control period	3.45 (1.99)	ref	21.78 (7.43)	ref	2.36 (1.57)	ref
Game with home nation	2.96 (2.18)	0.82 (0.65–1.05)	20.82 (5.03)	0.98 (0.93–1.03)	2.04 (1.79)	0.88 (0.67–1.15)
Other CS days	3.42 (1.97)	0.99 (0.90–1.09)	22.69 (5.98)	1.03 (1.00–1.06)	2.24 (1.53)	0.97 (0.88–1.08)
Women						
Control period	1.32 (1.24)	ref	8.29 (3.33)	ref	0.90 (0.94)	ref
Game with home nation	1.70 (1.22)	1.24 (0.90–1.72)	8.21 (3.50)	1.01 (0.92–1.10)	1.32 (0.94)	1.47 (1.05–2.05)
Other CS days	1.14 (1.09)	0.90 (0.77–1.06)	8.30 (3.32)	1.01 (0.96–1.06)	0.92 (1.02)	1.04 (0.88–1.22)
Aged 30–44						
Control period	1.00 (1.02)	ref	6.61 (3.19)	ref	0.70 (0.82)	ref
Game with home nation	0.57 (0.66)	0.55 (0.32–0.96)	6.39 (3.12)	1.00 (0.91–1.10)	0.68 (0.77)	1.04 (0.66–1.66)
Other CS days	1.02 (1.04)	1.04 (0.87–1.24)	6.81 (3.59)	1.05 (1.00–1.11)*	0.67 (0.84)	1.03 (0.85–1.24)
Men aged 15–34						
Control period	0.84 (1.00)	ref	4.00 (2.46)	ref	0.48 (0.68)	ref
Game with home nation	0.74 (0.81)	0.82 (0.50–1.33)	3.93 (2.23)	0.99 (0.88–1.12)	0.29 (0.53)	0.63 (0.31–1.27)
Other CS days	0.79 (0.89)	0.93 (0.77–1.14)	4.16 (2.49)	1.03 (0.96–1.10)	0.44 (0.63)	0.99 (0.78–1.24)

*Note:* IRRs are adjusted for day of week, month and year.

CS, championship; IRR, incidence risk ratios; ref, reference category.

* *P* < 0.10.

### Days during CS period when the home team won

In Germany, the incidence of suicide was significantly higher (IRR = 1.04; 95% CI: 1.01–1.07, *P *=* *0.016) on days when the home team did not play or played and lost, compared to the control period (Supplementary table S1). No statistically significant associations were found in any subgroup. However, in the subgroup analysis looking at the male German population, a non-significant statistical trend towards an increase of suicides on days during the CS other than the days that Germany won was found (IRR = 1.04; 95% CI: 1.01–1.07, *P *=* *0.052) (Supplementary table S1). Applying the sensitivity analysis, including the day following the home team’s victory, a non-significant statistical trend towards a higher rate of suicide among German men (IRR = 1.04; 95% CI: 1.01–1.08, *P *=* *0.056) and in Germany’s total sample (IRR = 1.04; 95% CI: 1.01–1.07, *P *=* *0.059) was detected on CS days other than the ones when the home team won (Supplementary table S1a). No statistically significant results were detected in the Austrian and Swiss population.

### Days during CS period when the home team lost

Incidences of suicides on days that the home team lost games vs. the control period vs. other CS days are presented in Supplementary table S2. In Austria and Switzerland, no significant differences were found between the days when the national team lost a game and the control period or other days of the CS in any of the studied subgroups. Compared to the control period, only a non-significant statistical trend towards a higher incidence of daily suicides on days when the home team lost was seen among Swiss women (IRR = 1.85; 95% CI: 1.17–2.94, *P *=* *0.056), but not in other subgroups (Supplementary table S2). However, this finding disappeared in the sensitivity analysis, which also included the next day (Supplementary table S2a). In Germany, during CS days other than the ones when the national team lost games, the incidence of daily suicides was significantly higher in the total sample and among men (both IRR = 1.05; 95% CI: 1.02–1.08, *P *<* *0.001). These findings remained statistically significant in the sensitivity analysis including the day following the game (Supplementary table S2a), while a similar trend was shown among people aged 30–44 years (IRR = 1.07; 95% CI: 1.02–1.13, *P *=* *0.076). There were no statistically significant differences in any other subgroups.

### Knock-out games

No statistically significant associations were found between daily suicide incidence during days when the national team participated in knock-out games vs. the control period vs. other CS days in all three studied nations and in the subgroups (Supplementary table S3). In Germany, however, during days of championships other than those when the national team played knock-out games, a non-significant statistical trend towards a higher incidence of daily suicides was registered in the total sample (IRR = 1.03; 95% CI: 1.01–1.06, *P *=* *0.084), but not in any of the subgroups.

### When Germany won the CS

The incidence of suicide on days that Germany won the CS was not statistically different from the rates during the control period including the following day (table 3).

**Table 3.** Incidences of daily suicides during days when Germany won the championship vs. control period vs. other championship days

	Only day of endgame		Including the next day	
	*M* (SD)	IRR (95% CI)	*M* (SD)	IRR (95% CI)
Total sample				
Control period	31.87 (9.36)	ref	31.81 (9.36)	ref
Won endgame	28.50 (4.95)	0.91 (0.70–1.18)	34.75 (7.80)	0.99 (0.83–1.17)
Other CS days	29.94 (7.53)	0.93 (0.88–0.99)	29.94 (7.53)	0.93 (0.88–0.99)
Men				
Control period	23.14(6.94)	ref	23.12 (6.96)	ref
Won endgame	21.00 (5.66)	0.92 (0.68–1.25)	23.75 (4.99)	0.93 (0.76–1.14)
Other CS days	21.85 (5.71)	0.94 (0.88–1.00)	21.85 (5.72)	0.94 (0.88–1.00)
Women				
Control Period	8.94(3.75)	ref	8.90 (3.72)	ref
Won endgame	7.50 (0.71)	0.89 (0.53–1.49)	11.00 (4.96)	1.19 (0.88–1.61)
Other CS days	8.09 (3.33)	0.94 (0.85–1.05)	8.09 (3.33)	0.95 (0.85–1.05)
Aged 30–44				
Control period	6.75 (3.37)	ref	6.73 (3.37)	ref
Won endgame	6.00 (1.41)	0.90 (0.51–1.60)	7.50 (3.11)	1.04 (0.72–1.50)
Other CS days	6.08 (4.61)	0.96 (0.85–1.09)	6.08 (4.61)	0.96 (0.85–1.09)
Men aged 30–44				
Control period	4.60 (2.78)	ref	4.59 (2.78)	ref
Won endgame	3.00 (2.83)	0.60 (0.27–1.35)	4.50 (2.52)	0.87 (0.54–1.40)
Other CS days	4.04 (2.49)	0.89 (0.77–1.03)	4.04 (2.49)	0.89 (0.77–1.03)

*Note:* IRRs are adjusted for day of week, month and year.

CS, championship; IRR, incidence risk ratios; ref, reference category.

#### The Wonder of Cordoba

During the day that Austria won against the then-reigning world champion Germany (colloquially referred to as ‘the Wonder of Cordoba’) on 21st June 1978, Austria’s incidence of suicide was not statistically different from the control period or other CS days, including the day following the game (Supplementary table S4).

## Discussion

Here, we show for the first time, in a large population-based cohort of three German-speaking countries, that there was no lowered suicide risk during major sports events, as would be expected from assumed increased social connectedness. We also did not detect changes in suicide rates depending on the outcome of important games as predicted by the broken promise effect or changes in self-efficacy by identification with winning teams. Consequently, such major sporting events are unlikely to affect suicide rates in the population at large.

To date, evidence for the association between major sporting events and their effect on suicide rates was inconclusive.^8^^,^^11^^,^^12^ In our study on changes in incidences of suicide during major soccer events in German-speaking countries, we expected—based on theoretical considerations, intuition and the existing literature—a positive effect of these events on suicide rates in the three studied nations, except when home teams lost. For example, we hypothesized that during CS periods there should be increased social connectedness (‘pulling together’) by feeling less lonely or increased social contact,^2^^,^^3^^,^^8^ resulting in lower suicide rates. Indeed, during and after the World Cup in 1990, Masterton and Mander observed a significant reduction in emergency psychiatric hospital presentations including parasuicidal behaviour of Scottish men and women,^19^ but no suicide rates were reported in this study.

In our study, we obtained mixed and partially unexpected results. There were essentially no statistically significant changes in the incidence of daily suicides during soccer championships compared to a control period. The few results that were significant or near significant suggest an increase in suicide rates during the CS period, but the effects were mostly small and the lower bounds of the CI were close to the null effect. However, in Germany, this increase was not observed on days when the German soccer team played (in general and in knock-out games). This could be shown irrespective of the team’s performance during the tournaments. Thus, perhaps the ‘pulling together’ effect during major sports events is smaller than assumed. Alternatively, the positive effect may be overshadowed by co-occurring elevated suicide-related risk factors such as increased alcohol consumption during soccer championships in the German-speaking regions. Durbeej et al. have already reported increased alcohol consumption using breath analysers among Swedish Premier Football League spectators, especially among young men located in a supporter section of a stadium.^20^ Although only being a statistically significant trend, a finding of potential concern in our study was that, in Switzerland, the results suggested that suicide rates increased among women when the Swiss national team lost a game. We could not observe such associations in Germany and Austria, nor in the sensitivity analysis—not even the day after the match—therefore, future studies with more data from Switzerland might be helpful to verify if this is a false positive finding. In line with our concerning finding in Switzerland, Hassanian-Moghaddam et al.^21^ previously reported an increase in hospitalizations in Teheran following intentional self-poisoning among females aged 12–20 years but not in males or other age groups during the 4-week period of World Cup 2014. An increase in violence towards women during major sporting events has been reported in other studies, too, and there seems to be a connection with increased alcohol use.^22^^,^^23^ In an attempt to explain this finding, White et al. argue that male spectators of sports events who watch their physically dominating team winning, which is again associated with positive emotions, might feel accredited and justified to use similar physical dominance in their home and against their partners to get what they want. Another well-known aspect in this context is the association between alcohol abuse and domestic violence, which can partially explain an increase in domestic violence during major sports events.^22^^,^^24^^,^^25^ A piece of evidence that is indirectly in line with a detrimental effect of major sports events on partnership quality is that an increase in national teams’ performance in international football competitions was associated with a drop in births 9 months after the event.^26^ These potential detrimental effects need to be explored in more detail in further studies.

Another hypothesis of our study was that we expected a lowered suicide incidence on days when the home team won, based on the assumption that identification with a winning team increases self-efficacy, a protective factor for suicide.^4^ In contrast, when home teams lose, self-efficacy may be reduced, and together with the ‘broken promise effect’, where positive expectations are diminished, suicide risk may be increased.^7^ However, the results were inconsistent. In all three countries, no significant changes in suicide rates were observed on days that the home team lost or won compared to the control period. However, in Germany, the incidence of suicides was higher during days of the CS period other than those when the home team played (won or lost); thus, it seems that on days when Germany played, the increase of suicide risk observed during the CS period was interrupted. Perhaps the detrimental effects associated with championships (e.g. increased alcohol consumption) are counterbalanced by suicide-protective factors (connectedness on days that the home team plays). Therefore, we could neither verify nor completely dismiss our hypothesis that a national team’s performance is related to a change in suicide incidence. Interestingly, even on the 6 days that the German national team won their championships, there was no significant change in the national suicide incidence. Notably, there was a numerical decrease in suicide rates (e.g. IRR 0.60); however, the CI was too wide and, thus, these results remain inconclusive with larger samples required. Similarly, there was no reduction in Austrian suicide rates during the ‘*The Wonder of Cordoba*’, the historically meaningful event for soccer-excited Austrians, when Austria won against Germany for the first and only time at a Soccer World Cup tournament (which would be comparable to the ‘Miracle on Ice’ when the US Hockey Team defeated the Russians). Therefore, in contrast to previous assumptions, the results of our study do not support the assumption that suicide rates could be influenced by either the vicarious self-efficacy effect or the broken promise effect.

Generally, we expected that the assumed effects could be most pronounced in Germany, where soccer is very popular, and because Germany is the most successful of these three nations in international soccer championships. However, our results do not confirm this assumption. Although Germany was the only studied nation where we found certain trends and even significant associations between the suicide rates and soccer events, none of these associations was related to the performance of the national team. We also expected that the effects would be most pronounced in younger males who perhaps identify themselves more with soccer, the fan culture, and who are more likely to meet with other fans. Therefore, they might be more vulnerable to the emotional effects of a soccer game, which is subsequently reflected in the national suicide rate. Indeed, in a French study exploring this hypothesis for the FIFA World Cup 1998, a significant decline of 95 suicides was observed (−10.3%); this effect was the strongest among men and people aged between 30 and 44.^11^ However, these findings could not be replicated in our study. None of the sex- and age-specific subgroup analyses revealed significant results. French and Hungarian studies were only based on single sporting events,^12^ but replicating the analysis with more tournaments in these countries may be interesting. Our results suggest that the effects of major sporting events discussed in suicidology should not be overestimated.

There are several limitations to this study. Although our sample was powered to detect small effects for the CS periods across the three nations, statistical power may have been too low to detect small effects in certain subgroup analyses or for the analysis of single days. Thus, some of our findings may have occurred by chance, whereas some results may become statistically significant in larger samples. Some potential confounding variables, such as alcohol consumption or weather condition remained unassessed, as did direct measures of moderating variables (e.g. self-efficacy, social connectedness). Future studies could control for these confounding and moderating factors, or even use real-time monitoring of suicide-related risk and protective factors among at-risk populations during major sporting events.

In conclusion, we did not find significant reductions in the incidence of daily suicides during soccer championships in Austria, Germany and Switzerland in contrast to the hypothesis of increased social connectedness and thus lowered suicide risk during major sporting events. Furthermore, days with a winning or losing home team were not associated with changes in suicide incidences, which contrasts with predictions based on the broken promise effect or changes in self-efficacy by identification with winning teams. Methodological limitations call for replication in other countries and using other research designs.

## Supplementary Material

ckad042_Supplementary_Data

## Data Availability

All data supporting the results of this study, including the results of the sensitivity analyses and the R-Code, are available on Open Science Framework (https://osf.io/4b6t5/). ‘Increased social connectedness’, ‘self-efficacy’ and the ‘broken promise effect’ are well-known concepts in suicidology, but their reference to sporting events and relating suicide rates remain inconclusive. In our study, soccer championships were not associated with changes of suicide rates expected from postulated psychological effects among spectators. No specific subgroup of the general population in Austria, Germany or Switzerland appeared to be particularly vulnerable to suicide during major soccer championships. Limitations: Potentially important confounding factors such as alcohol consumption were not considered in the analyses and direct measures of the putative psychological mechanisms remain unassessed.
