# Supplementary material for: Alive and kicking: suicide rates and major soccer events in Austria, Germany and Switzerland
Source: Eur J Public Health. 2023 Mar 20;33(3):424–9. doi: 10.1093/eurpub/ckad042 (PMC10234653; doi:10.1093/eurpub/ckad042)
Supplement: ckad042_Supplementary_Data [file ckad042_supplementary_data.zip › ckad042_Supplementary_Data/ejph-2023-01-om-0010-File003.doc]

Supplement Table 1. Incidences of daily suicides during days **when the home team won** games versus control period versus other championship days.

|  | Austria | | | | Germany | | | | Switzerland | | | |
| --- | --- | --- | --- | --- | --- | --- | --- | --- | --- | --- | --- | --- |
|  | M | (SD) | IRR | (95% CI) | M | (SD) | IRR | (95% CI) | M | (SD) | IRR | (95% CI) |
| Total Sample |  |  |  |  |  |  |  |  |  |  |  |  |
| Control period | 5.57 | (2.38) | ref |  | 29.50 | (10.27) | ref |  | 3.08 | (1.75) | ref |  |
| Games won | 3.83 | (2.64) | 0.67 | (0.44-1.02) | 28.66 | (5.62) | 0.98 | (0.92-1.04) | 2.62 | (1.51) | 0.86 | (0.56-1.33) |
| Other CS days | 5.29 | (2.45) | 0.95 | (0.86-1.06) | 31.09 | (7.64) | 1.04 | (1.01-1.07)^*^ | 3.06 | (1.90) | 1.01 | (0.92-1.11) |
|  |  |  |  |  |  |  |  |  |  |  |  |  |
| Men |  |  |  |  |  |  |  |  |  |  |  |  |
| Control Period | 3.91 | (1.96) | ref |  | 21.60 | (7.70) | ref |  | 2.24 | (1.49) | ref |  |
| Game won | 2.00 | (1.90) | 0.50 | (0.28-0.88) | 20.84 | (4.64) | 0.97 | (0.91-1.04) | 1.38 | (1.30) | 0.63 | (0.34-1.14) |
| Other CS days | 3.90 | (2.15) | 0.99 | (0.87-1.12) | 22.71 | (6.08) | 1.04 | (1.01-1.07) ^+^ | 2.14 | (1.54) | 0.98 | (0.87-1.10) |
|  |  |  |  |  |  |  |  |  |  |  |  |  |
| Women |  |  |  |  |  |  |  |  |  |  |  |  |
| Control Period | 1.66 | (1.30) | ref |  | 8.33 | (3.41) | ref |  | 0.84 | (0.92) | ref |  |
| Games won | 1.83 | (1.60) | 1.10 | (0.60-2.01) | 7.82 | (3.57) | 0.97 | (0.87-1.08) | 1.25 | (0.71) | 1.47 | (0.78-2.76) |
| Other CS days | 1.39 | (1.17) | 0.87 | (0.71-1.06) | 8.37 | (3.30) | 1.01 | (0.96-1.06) | 0.92 | (1.01) | 1.10 | (0.93-1.31) |
|  |  |  |  |  |  |  |  |  |  |  |  |  |
| Aged 30-44 |  |  |  |  |  |  |  |  |  |  |  |  |
| Control Period | 1.19 | (1.06) | ref |  | 6.42 | (3.20) | ref |  | 0.65 | (0.79) | ref |  |
| Games won | 0.33 | (0.52) | 0.28 | (0.07-1.14) | 6.30 | (3.11) | 1.02 | (0.90-1.15) | 0.38 | (0.52) | 0.63 | (0.20-1.98) |
| Other CS days | 1.20 | (1.05) | 1.01 | (0.80-1.26) | 6.74 | (3.71) | 1.07 | (1.01-1.12) | 0.68 | (0.84) | 1.09 | (0.89-1.34) |
|  |  |  |  |  |  |  |  |  |  |  |  |  |
| Men, aged 15-34 |  |  |  |  |  |  |  |  |  |  |  |  |
| Control Period | 1.07 | (1.08) | ref |  | 3.99 | (2.54) | ref |  | 0.43 | (0.65) | ref |  |
| Games won | 0.67 | (1.03) | 0.62 | (0.23-1.68) | 3.86 | (2.46) | 0.97 | (0.83-1.14) | 0.25 | (0.46) | 0.62 | (0.15-2.52) |
| Other CS days | 1.04 | (0.93) | 0.94 | (0.74-1.19) | 4.21 | (2.51) | 1.04 | (0.97-1.11) | 0.37 | (0.59) | 0.92 | (0.70-1.20) |

CS: Championship; IRR: Incidence risk ratios; ref: reference category; IRRs are adjusted for day of week, month, and year; ^+^p<0.10; *p < 0.05; **p < 0.01
