# Supplementary material for: Alive and kicking: suicide rates and major soccer events in Austria, Germany and Switzerland
Source: Eur J Public Health. 2023 Mar 20;33(3):424–9. doi: 10.1093/eurpub/ckad042 (PMC10234653; doi:10.1093/eurpub/ckad042)
Supplement: ckad042_Supplementary_Data [file ckad042_supplementary_data.zip › ckad042_Supplementary_Data/ejph-2023-01-om-0010-File004.doc]

Supplement Table 1a. Sensitivity Analysis. Incidences of daily suicides during days **when the home team won** games (including the next day) versus control period versus other championship days.

|  | Austria | | | | Germany | | | | Switzerland | | | |
| --- | --- | --- | --- | --- | --- | --- | --- | --- | --- | --- | --- | --- |
|  | M | (SD) | IRR | (95% CI) | M | (SD) | IRR | (95% CI) | M | (SD) | IRR | (95% CI) |
| Total Sample |  |  |  |  |  |  |  |  |  |  |  |  |
| Control period | 5.57 | (2.38) | ref |  | 29.48 | (10.27) | ref |  | 3.08 | (1.75) | ref |  |
| Game won +1d | 5.25 | (3.52) | 0.93 | (0.72-1.20) | 30.48 | (7.81) | 1.02 | (0.98-1.06) | 2.56 | (1.71) | 0.83 | (0.60-1.13) |
| Other CS days | 5.18 | (2.29) | 0.94 | (0.83-1.04) | 30.94 | (7.31) | 1.04 | (1.01-1.07) ^+^ | 3.09 | (1.89) | 1.02 | (0.93-1.13) |
|  |  |  |  |  |  |  |  |  |  |  |  |  |
| Men |  |  |  |  |  |  |  |  |  |  |  |  |
| Control Period | 3.91 | (1.96) | ref |  | 21.59 | (7.70) | ref |  | 2.24 | (1.49) | ref |  |
| Game won +1d | 3.67 | (2.96) | 0.92 | (0.68-1.25) | 21.89 | (5.52) | 1.00 | (0.95-1.05) | 1.69 | (1.58) | 0.75 | (0.51-1.10) |
| Other CS days | 3.78 | (2.04) | 0.96 | (0.84-1.09) | 22.71 | (6.07) | 1.04 | (1.01-1.08) ^+^ | 2.15 | (1.53) | 0.98 | (0.88-1.10) |
|  |  |  |  |  |  |  |  |  |  |  |  |  |
| Women |  |  |  |  |  |  |  |  |  |  |  |  |
| Control Period | 1.66 | (1.30) | ref |  | 8.32 | (3.40) | ref |  | 0.84 | (0.92) | ref |  |
| Game won +1d | 1.58 | (1.38) | 0.96 | (0.61-1.53) | 8.59 | (4.18) | 1.05 | (0.97-1.13 | 0.88 | (0.72) | 1.02 | (0.60-1.75) |
| Other CS days | 1.40 | (1.18) | 0.87 | (0.70-1.07) | 8.23 | (3.01) | 1.00 | (0.95-1.05) | 0.94 | (1.02) | 1.13 | (0.95-1.35) |
|  |  |  |  |  |  |  |  |  |  |  |  |  |
| Aged 30-44 |  |  |  |  |  |  |  |  |  |  |  |  |
| Control Period | 1.19 | (1.06) | ref |  | 6.42 | (3.20) | ref |  | 0.65 | (0.79) | ref |  |
| Game won +1d | 1.08 | (1.24) | 0.92 | (0.53-1.61) | 6.80 | (3.78) | 1.09 | (1.00-1.18) | 0.44 | (0.63) | 072 | (0.34-1.53) |
| Other CS days | 1.15 | (1.02) | 0.96 | (0.76-1.21) | 6.65 | (3.57) | 1.05 | (0.99-1.11) | 0.69 | (0.84) | 1.11 | (0.90-1.36) |
|  |  |  |  |  |  |  |  |  |  |  |  |  |
| Men, aged 15-34 |  |  |  |  |  |  |  |  |  |  |  |  |
| Control Period | 1.07 | (1.08) | ref |  | 3.98 | (2.54) | ref |  | 0.43 | (0.65) | ref |  |
| Game won +1d | 1.33 | (1.37) | 1.25 | (0.75-2.07) | 4.05 | (2.46) | 1.01 | (0.90-1.13) | 0.31 | (0.60) | 0.76 | (0.31-1.86) |
| Other CS days | 0.96 | (0.84) | 0.87 | (0.67-1.12) | 4.22 | (2.51) | 1.04 | (0.96-1.12) | 0.37 | (0.59) | 0.92 | (0.70-1.21) |

CS: Championship; IRR: Incidence risk ratios; ref: reference category; IRRs are adjusted for day of week, month, and year; ^+^p<0.10; *p < 0.05; **p < 0.01
