# Supplementary material for: Alive and kicking: suicide rates and major soccer events in Austria, Germany and Switzerland
Source: Eur J Public Health. 2023 Mar 20;33(3):424–9. doi: 10.1093/eurpub/ckad042 (PMC10234653; doi:10.1093/eurpub/ckad042)
Supplement: ckad042_Supplementary_Data [file ckad042_supplementary_data.zip › ckad042_Supplementary_Data/ejph-2023-01-om-0010-File005.doc]

Supplement Table 2. Incidences of daily suicides during days **when the home team lost** games versus control period versus other championship days.

|  | Austria | | | | Germany | | | | Switzerland | | | |
| --- | --- | --- | --- | --- | --- | --- | --- | --- | --- | --- | --- | --- |
|  | M | (SD) | IRR | (95% CI) | M | (SD) | IRR | (95% CI) | M | (SD) | IRR | (95% CI) |
| Total Sample |  |  |  |  |  |  |  |  |  |  |  |  |
| Control period | 4.76 | (2.44) | ref |  | 29.30 | (9.83) | ref |  | 3.37 | (1.93) | ref |  |
| Games lost | 5.33 | (3.03) | 1.10 | (0.85-1.41) | 28.27 | (6.03) | 1.02 | (0.93-1.13) | 3.82 | (2.56) | 1.12 | (0.82-1.53) |
| Other CS days | 4.52 | (2.27) | 0.95 | (0.88-1.04) | 30.84 | (7.43) | 1.05 | (1.02-1.08)^**^ | 3.34 | (1.98) | 1.01 | (0.92-1.12) |
|  |  |  |  |  |  |  |  |  |  |  |  |  |
| Men |  |  |  |  |  |  |  |  |  |  |  |  |
| Control Period | 3.45 | (1.99) | ref |  | 21.52 | (7.50) | ref |  | 2.45 | (1.62) | ref |  |
| Game lost | 3.58 | (2.39) | 1.02 | (0.75-1.38) | 19.60 | (4.91) | 0.97 | (0.86-1.09) | 2.09 | (2.43) | 0.85 | (0.56-1.28) |
| Other CS days | 3.34 | (1.97) | 0.97 | (0.88-1.06) | 22.54 | (5.90) | 1.05 | (1.02-1.08)^**^ | 2.32 | (1.61) | 0.97 | (0.87 1.09) |
|  |  |  |  |  |  |  |  |  |  |  |  |  |
| Women |  |  |  |  |  |  |  |  |  |  |  |  |
| Control Period | 1.32 | (1.24) | ref |  | 8.16 | (3.22) | ref |  | 0.92 | (0.95) | ref |  |
| Games lost | 1.75 | (1.22) | 1.31 | (0.84-2.03) | 8.67 | (3.04) | 1.07 | (0.90-1.28) | 1.73 | (1.01) | 1.85 | (1.17-2.94) ^+^ |
| Other CS days | 1.18 | (1.11) | 0.92 | (0.79-1.08) | 8.30 | (3.39) | 1.02 | (0.97-1.07) | 1.02 | (1.04) | 1.12 | (0.94-1.34) |
|  |  |  |  |  |  |  |  |  |  |  |  |  |
| Aged 30-44 |  |  |  |  |  |  |  |  |  |  |  |  |
| Control Period | 1.00 | (1.02) | ref |  | 6.58 | (3.16) | ref |  | 0.71 | (0.84) | ref |  |
| Games lost | 0.67 | (0.65) | 0.66 | (0.33-1.34) | 6.33 | (3.04) | 0.99 | (0.81-1.22) | 0.82 | (0.98) | 1.17 | (0.60-2.29) |
| Other CS days | 0.98 | (1.02) | 0.99 | (0.83-1.18) | 6.88 | (3.29) | 1.06 | (1.01-1.12) | 0.72 | (0.86) | 1.08 | (0.88-1.33) |
|  |  |  |  |  |  |  |  |  |  |  |  |  |
| Men, aged 15-34 |  |  |  |  |  |  |  |  |  |  |  |  |
| Control Period | 0.84 | (1.00) | ref |  | 3.89 | (2.39) | ref |  | 0.51 | (0.71) | ref |  |
| Games lost | 1.00 | (0.74) | 1.12 | (0.63-1.99) | 4.27 | (1.71) | 1.15 | (0.89-1.47) | 0.27 | (0.65) | 0.55 | (0.17-1.73) |
| Other CS days | 0.77 | (0.88) | 0.90 | (0.74-1.10) | 4.13 | (2.47) | 1.05 | (0.98-1.12) | 0.53 | (0.66) | 1.11 | (0.87-1.41) |

CS: Championship; IRR: Incidence risk ratios; ref: reference category, IRRs are adjusted for day of week, month, and year. ^+^p<0.10; *p < 0.05; **p < 0.01
