# Supplementary material for: Alive and kicking: suicide rates and major soccer events in Austria, Germany and Switzerland
Source: Eur J Public Health. 2023 Mar 20;33(3):424–9. doi: 10.1093/eurpub/ckad042 (PMC10234653; doi:10.1093/eurpub/ckad042)
Supplement: ckad042_Supplementary_Data [file ckad042_supplementary_data.zip › ckad042_Supplementary_Data/ejph-2023-01-om-0010-File006.doc]

Supplement Table 2a. Sensitivity Analysis. Incidences of daily suicides during days **when the home team lost** games (including the next day) versus control period versus other championship days.

|  | Austria | | | | Germany | | | | Switzerland | | | |
| --- | --- | --- | --- | --- | --- | --- | --- | --- | --- | --- | --- | --- |
|  | M | (SD) | IRR | (95% CI) | M | (SD) | IRR | (95% CI) | M | (SD) | IRR | (95% CI) |
| Total Sample |  |  |  |  |  |  |  |  |  |  |  |  |
| Control period | 4.76 | (2.44) | ref |  | 29.30 | (9.29) | ref |  | 3.37 | (1.93) | ref |  |
| Games lost +1d | 4.96 | (2.39) | 1.01 | (0.84-1.22) | 28.57 | (6.23) | 1.02 | (0.95-1.09) | 3.36 | (2.17) | 0.99 | (0.78-1.25) |
| Other CS days | 4.51 | (2.32) | 0.96 | (0.88-1.04) | 30.96 | (7.47) | 1.05 | (1.03-1.08)^**^ | 3.38 | (2.00) | 1.03 | (0.93-1.13) |
|  |  |  |  |  |  |  |  |  |  |  |  |  |
| Men |  |  |  |  |  |  |  |  |  |  |  |  |
| Control Period | 3.45 | (1.99) | ref |  | 21.52 | (7.50) | ref |  | 2.45 | (1.62) | ref |  |
| Game lost +1d | 3.50 | (2.04) | 0.98 | (0.79-1.23) | 20.43 | (4.84) | 0.99 | (0.91-1.08) | 2.14 | (2.01) | 0.87 | (0.65-1.16) |
| Other CS days | 3.34 | (2.00) | 0.97 | (0.88-1.07) | 22.60 | (5.95) | 1.05 | (1.02-1.08) ^**^ | 2.33 | (1.61) | 0.98 | (0.87-1.10) |
|  |  |  |  |  |  |  |  |  |  |  |  |  |
| Women |  |  |  |  |  |  |  |  |  |  |  |  |
| Control Period | 1.32 | (1.24) | ref |  | 8.16 | (3.23) | ref |  | 0.92 | (0.95) | ref |  |
| Game lost +1d | 1.46 | (1.06) | 1.09 | (0.77-1.53) | 8.13 | (3.05) | 0.99 | (0.87-1.13) | 1.23 | (1.11) | 1.31 | (0.89-1.94) |
| Other CS days | 1.18 | (1.13) | 0.93 | (0.79-1.09) | 8.36 | (3.41) | 1.03 | (0.98-1.08) | 1.04 | (1.04) | 1.15 | (0.96-1.38) |
|  |  |  |  |  |  |  |  |  |  |  |  |  |
| Aged 30-44 |  |  |  |  |  |  |  |  |  |  |  |  |
| Control Period | 1.00 | (1.02) | ref |  | 6.58 | (3.16) | ref |  | 0.71 | (0.84) | ref |  |
| Game lost +1d | 0.92 | (1.06) | 0.91 | (0.59-1.40) | 6.07 | (2.99) | 0.94 | (0.81-1.09) | 0.64 | (0.79) | 0.91 | (0.53-1.56) |
| Other CS days | 0.97 | (1.00) | 0.98 | (0.82-1.17) | 6.93 | (3.29) | 1.07 | (1.02-1.13) ^+^ | 0.74 | (0.88) | 1.11 | (0.90-1.37) |
|  |  |  |  |  |  |  |  |  |  |  |  |  |
| Men aged 15-34 |  |  |  |  |  |  |  |  |  |  |  |  |
| Control Period | 0.84 | (1.00) | ref |  | 3.88 | (2.39) | ref |  | 0.51 | (0.71) | ref |  |
| Game lost +1d | 0.92 | (0.93) | 1.03 | (0.67 -1.58) | 4.13 | (1.72) | 1.10 | (0.92-1.32) | 0.36 | (0.66) | 0.73 | (0.36-1.49) |
| Other CS days | 0.76 | (0.87) | 0.90 | (0.73 -1.10) | 4.15 | (2.49) | 1.06 | (0.99-1.13) | 0.53 | (0.66) | 1.12 | (0.87-1.44) |

CS: Championship; IRR: Incidence risk ratios; ref: reference category, IRRs are adjusted for day of week, month, and year. ^+^p<0.10; *p < 0.05; **p < 0.01
