# Supplementary material for: Alive and kicking: suicide rates and major soccer events in Austria, Germany and Switzerland
Source: Eur J Public Health. 2023 Mar 20;33(3):424–9. doi: 10.1093/eurpub/ckad042 (PMC10234653; doi:10.1093/eurpub/ckad042)
Supplement: ckad042_Supplementary_Data [file ckad042_supplementary_data.zip › ckad042_Supplementary_Data/ejph-2023-01-om-0010-File007.doc]

Supplement Table 3. Incidences of daily suicides during days when the home nation was in **knock-out games** versus control period versus other championship days.

|  | Austria | | | | Germany | | | | Switzerland | | | |
| --- | --- | --- | --- | --- | --- | --- | --- | --- | --- | --- | --- | --- |
|  | M | (SD) | IRR | (95% CI) | M | (SD) | IRR | (95% CI) | M | (SD) | IRR | (95% CI) |
| Total Sample |  |  |  |  |  |  |  |  |  |  |  |  |
| Control period | 5.62 | (2.45) | ref |  | 29.50 | (10.27) | ref |  | 3.18 | (1.82) | ref |  |
| Knock-out games | 6.00 | (0.71) | 1.05 | (0.73-1.51) | 28.49 | (5.12) | 1.02 | (0.96-1.09) | 4.25 | (2.06) | 1.40 | (0.86-2.26) |
| Other CS days | 5.47 | (2.67) | 0.97 | (0.85-1.11) | 31.03 | (7.63) | 1.03 | (1.01-1.06) ^+^ | 2.98 | (1.97) | 0.96 | (0.85-1.07) |
|  |  |  |  |  |  |  |  |  |  |  |  |  |
| Men |  |  |  |  |  |  |  |  |  |  |  |  |
| Control Period | 3.98 | (1.98) | ref |  | 21.60 | (7.70) | ref |  | 2.30 | (1.57) | ref |  |
| Knock-out games | 3.80 | (0.45) | 0.93 | (0.59-1.47) | 20.86 | (4.42) | 1.02 | (0.95-1.10) | 3.25 | (1.71) | 1.48 | (0.85-2.57) |
| Other CS days | 4.02 | (2.36) | 1.00 | (0.86-1.16) | 22.65 | (6.07) | 1.03 | (1.00-1.06) | 2.13 | (1.51) | 0.95 | (0.83-1.09) |
|  |  |  |  |  |  |  |  |  |  |  |  |  |
| Women |  |  |  |  |  |  |  |  |  |  |  |  |
| Control Period | 1.64 | (1.30) | ref |  | 8.33 | (3.41) | ref |  | 0.88 | (0.93) | ref |  |
| Knock-out games | 2.20 | (0.84) | 1.35 | (0.74-2.48) | 7.63 | (3.60) | 0.93 | (0.82-1.05) | 1.00 | (0.82) | 1.18 | (0.44-3.19) |
| Other CS days | 1.45 | (1.37) | 0.91 | (0.70-1.17) | 8.38 | (3.31) | 1.02 | (0.97-1.07) | 0.84 | (1.00) | 0.97 | (0.78-1.20) |
|  |  |  |  |  |  |  |  |  |  |  |  |  |
| Aged 30-44 |  |  |  |  |  |  |  |  |  |  |  |  |
| Control Period | 1.22 | (1.09) | ref |  | 6.42 | (3.20) | ref |  | 0.67 | (0.81) | ref |  |
| Knock-out games | 0.80 | (0.84) | 0.65 | (0.24-1.75) | 6.00 | (2.93) | 0.96 | (0.84-1.11) | 1.00 | (0.82) | 1.59 | (0.59-4.30) |
| Other CS days | 1.24 | (1.05) | 1.01 | (0.77-1.34) | 6.76 | (3.70) | 1.07 | (1.02-1.13) | 0.64 | (0.83) | 1.00 | (0.78-1.28) |
|  |  |  |  |  |  |  |  |  |  |  |  |  |
| Males aged 30-44 |  |  |  |  |  |  |  |  |  |  |  |  |
| Control Period | 1.04 | (1.06) | ref |  | 3.98 | (2.54) | ref |  | 0.47 | (0.68) | ref |  |
| Knock-out games | 1.40 | (0.55) | 1.32 | (0.62-2.81) | 4.20 | (1.95) | 1.09 | (0.93-1.29) | 0 | (0) | 0.00 | (0.00) |
| Other CS days | 1.04 | (1.06) | 0.95 | (0.70-1.29) | 4.15 | (2.56) | 1.02 | (0.95-1.09) | 0.36 | (0.61) | 0.82 | (0.60-1.14) |

CS: Championship; IRR: Incidence risk ratios; ref: reference category; IRRs are adjusted for day of week, month, and year. ^+^p<0.10; *p < 0.05; **p < 0.01
