# Supplementary material for: Alive and kicking: suicide rates and major soccer events in Austria, Germany and Switzerland
Source: Eur J Public Health. 2023 Mar 20;33(3):424–9. doi: 10.1093/eurpub/ckad042 (PMC10234653; doi:10.1093/eurpub/ckad042)
Supplement: ckad042_Supplementary_Data [file ckad042_supplementary_data.zip › ckad042_Supplementary_Data/ejph-2023-01-om-0010-File008.doc]

Supplement Table 4. The wonder of Cordoba – Austria beats Germany

|  | Day of Austria`s victory against Germany | | | | Including Next Day | | | |
| --- | --- | --- | --- | --- | --- | --- | --- | --- |
|  | M | (SD) | IRR | (95% CI) | M | (SD) | IRR | (95% CI) |
| Total Sample |  |  |  |  |  |  |  |  |
| Control period | 5.20 | (2.19) | ref |  | 5.20 | (2.19) | ref |  |
| Austria won | 6 | - | 1.14 | (0.51-2.56) | 6.50 | (0.71) | 1.26 | (0.72-2.19) |
| Other CS days | 5.46 | (2.57) | 1.05 | (0.87-1.27) | 5.39 | (2.61) | 1.04 | (0.86-1.26) |
|  |  |  |  |  |  |  |  |  |
| Men |  |  |  |  |  |  |  |  |
| Control Period | 3.31 | (1.26) | ref |  | 3.66 | (1.75) | ref |  |
| Austria won | 4.00 | - | 1.06 | (0.39-2.87) | 4.50 | (0.71) | 1.21 | (0.62-2.35) |
| Other CS days | 3.92 | (2.26) | 1.06 | (0.85-1.32) | 3.87 | (2.30) | 1.05 | (0.83-1.32) |
|  |  |  |  |  |  |  |  |  |
| Women |  |  |  |  |  |  |  |  |
| Control Period | 1.54 | (1.26) | ref |  | 1.54 | (1.26) | ref |  |
| Austria won | 2.00 | - | 1.33 | (0.32-5.42) | 2 | (0.00) | 1.38 | (0.51-3.76) |
| Other CS days | 1.54 | (1.38) | 1.04 | (0.73-1.48) | 1.52 | (1.41) | 1.05 | (0.71-1.47) |
|  |  |  |  |  |  |  |  |  |
| Aged 30-44 |  |  |  |  |  |  |  |  |
| Control Period | 1.14 | (1.13) | ref |  | 1.14 | (1.13) | ref |  |
| Austria won | 0.00 | - | 0.00 | (0.00) | 0.50 | (0.71) | 0.45 | (0.06-3.25) |
| Other CS days | 0.92 | (0.93) | 0.80 | (0.51-1.26) | 0.91 | (0.95) | 0.80 | (0.50-1.27) |
|  |  |  |  |  |  |  |  |  |
| Men aged 30-44 |  |  |  |  |  |  |  |  |
| Control Period | 0.99 | (0.99) | ref |  | 0.99 | (0.99) | ref |  |
| Austria won | 1.00 | (2.00) | 2.04 | (0.55-8.37) | 1.50 | (0.71) | 1.54 | (0.48-4.90) |
| Other CS days | 0.92 | (0.93) | 0.88 | (0.55-1.38) | 0.91 | (0.95) | 0.87 | (0.55-1.38) |

CS: Championship; IRR: Incidence risk ratios; ref: reference category; IRRs are adjusted for day of week, month, and year.

^+^p<0.10; *p < 0.05; **p < 0.01
